# Editorial: Animal communication: neurobiological, chemical, and physiological aspects

**DOI:** 10.3389/fvets.2026.1975048

**Published:** 2026-09-21

**Authors:** Daniel Mota-Rojas, Temple Grandin

**Affiliations:** 1 Neurophysiology, Behavior and Animal Welfare Assessment, DPAA, Universidad Autónoma Metropolitana (UAM), Mexico City, Mexico; 2 Department of Animal Science, Colorado State University, Fort Collins, CO, United States

**Keywords:** affective states, animal welfare, computational ethology, sensory biology, social signaling

## 1 Introduction

Animal communication underlies many of the interactions through which animals acquire information, coordinate social behavior, avoid predators, compete for resources, and reproduce successfully. Since the foundations of modern ethology were established, communication has been interpreted within a broader biological framework in which behavior is understood through its causation, development, function, and evolutionary history (1). Building on this foundation, evolutionary theories of animal signaling established that communication is shaped by natural selection through the dynamic interaction between signal production and receiver responses, both of which contribute to the evolution and maintenance of signaling systems (2, 3). This conceptual framework was further refined by recognizing that communication emerges from the interaction between signalers and receivers, whose behavioral responses actively determine the biological significance of signals (4). Complementing this perspective, communication has also been viewed as a process through which signals influence receiver behavior, extending classical information-based interpretations of animal signaling (5). Behavioral ecology, cognitive biology, and comparative research now examine animal communication in relation to the conditions under which signals are exchanged. Interactions between signalers and receivers are interpreted within their social and ecological settings, including relevant environmental conditions (6, 7). Neurobiological, physiological, and endocrine mechanisms also modulate signal production and perception. Endocrine mechanisms, in particular, can modify both functions in a context-dependent manner, linking an animal's internal physiological state to its responses across different signaling modalities (8, 9). Current research on animal communication therefore considers the mechanisms involved in the production, perception, and response to biologically relevant signals within their physiological and environmental context. The perception and exchange of biologically relevant signals are also relevant to animal welfare

assessment. Behavioral responses depend on sensory perception, internal physiological state, affective processes, and environmental conditions. Facial expressions have been examined as non-verbal indicators of affective states. Mota-Rojas et al. (10) reviewed the neurobiological basis linking emotional processing to facial expression in non-human mammals, noting that the neural pathways involved and the correspondence between specific facial movements and emotional states remain incompletely understood.

Interpretation of animal behavior depends substantially on how each species perceives and processes its environment. Grandin and Shivley (11) described how species-specific sensory characteristics and fearfulness influence responses to handling, restraint, and transport. Broom (12) defined welfare as the state of an individual in relation to its attempts to cope with its environment. Dawkins (13) argued that suffering in non-human animals can be investigated scientifically despite the lack of direct access to subjective experience and proposed examining both animal health and what animals seek or avoid. The Five Domains Model considers nutrition, the physical environment, health, and behavioral interactions as functional domains contributing to mental state and explicitly incorporates human–animal interactions (14).

Analysis of the biological mechanisms involved in communication also requires consideration of the neural and physiological processes that contribute to affective states and regulate behavioral responses. Panksepp (15) developed affective neuroscience as a psychobiological approach to investigating emotional systems in the mammalian brain (16). Koolhaas et al. (17) argued that physiological activation alone does not constitute stress and proposed reserving the term for situations in which environmental demands exceed an organism's natural regulatory capacity, particularly under conditions of unpredictability or lack of control. Mendl et al. (18) reviewed cognitive bias as a potential indicator of animal emotion and welfare, examining how affective states may alter information processing and judgement of ambiguous stimuli.

This Research Topic examines signal production, perception, and interpretation across species and experimental contexts, and their relationships with neural and endocrine regulation, physiology, behavior, and environmental conditions. Its scope includes sensory and social communication, affective states, including pain, physiological and environmental responses, and analytical methods applied to animal behavior and welfare. The studies span levels of biological organization from neural and endocrine mechanisms to behavioral expression and ecological context, and include questions relevant to animal welfare and veterinary science.

## 2 Communication, sensory biology and social signaling

Animal communication involves the production, perception, and interpretation of signals within specific sensory, physiological, and social contexts. The studies included in this Research Topic address these processes through different sensory modalities and biological settings, including olfactory and acoustic signaling, visual perception, maternal interactions, and social communication in domestic and wild species. Within this range of topics, several contributions examine olfaction because of its role in social and ecological interactions. Schild and Rørvang reviewed current knowledge of porcine olfaction, emphasizing that pigs possess a highly developed olfactory system that is functional from birth and participates in exploratory, social, reproductive, and feeding-related behavior. Their review further identifies important gaps in our understanding of behavioral responses to specific odorants and discusses the potential use of olfactory stimuli in commercial production systems, while acknowledging that these knowledge gaps may themselves have implications for animal welfare.

A similar reliance on chemical communication is evident in canids, although its expression appears to depend on biological context. Studer et al. experimentally exposed captive wolves to unfamiliar conspecific and heterospecific urine and found that breeding individuals displayed higher investigatory, territorial, and locomotor responses to conspecific odor than non-breeders. Rather than indicating a uniform behavioral response, these findings suggest that olfactory communication is influenced by breeding status and are consistent with an important role of conspecific scent in territorial behavior. Evidence from domestic dogs extends this perspective to interspecific communication. Capitain et al. examined dogs exposed to human fear and neutral chemosignals while preventing the accompanying experimenter from perceiving the odors. Dogs exposed to the fear-chemosignal condition remained closer to the experimenter, showed lower tail posture, and required more time to approach the odor targets, but did not display a group-level preference or avoidance of either target. The marked inter-individual variability observed led the authors to caution against interpreting these responses as evidence of an innate fear-avoidance mechanism.

Communication is likewise shaped by the way animals perceive visual information from their environment. Roth and McGreevy reviewed equine vision using Tinbergen's Four Questions together with the Five Domains framework. Their synthesis describes the anatomical and functional characteristics of the equine visual system and relates these features to behavioral responses, management practices, human–horse interactions, and welfare. The review indicates that interpretation of equine behavior should take into account the visual adaptations characteristic of a prey species, particularly when assessing responses to environmental or human-derived stimuli. Acoustic communication is another modality addressed in this Research Topic. Lenner et al. analyzed vocalizations produced by *Bos taurus* and *Bubalus bubalis* dams following temporary separation from their calves. Quantitative acoustic analysis revealed interspecific differences in parameters such as pitch dynamics and mel-frequency cepstral coefficients, whereas no clear individual differences were detected under the conditions evaluated.

The authors suggest that these acoustic features may support future applications in livestock bioacoustics. Acoustic communication was also examined in a different biological context by Hradec et al., who investigated the influence of hormonal contraception on vocal communication in captive southern yellow-cheeked gibbons. Longitudinal recordings showed changes in components of female great-call structure associated with contraceptive treatment, with responses differing according to contraceptive method and not always returning to pretreatment values after treatment effects waned. Male coda vocalizations, however, did not exhibit comparable structural adjustments.

Beyond sensory perception, communication also depends on the dynamic exchange of behavioral signals between individuals. Heberlein et al. examined gaze alternation and other showing behaviors in dogs under conditions that differed in the human partner's knowledge or cooperativeness. Dogs displayed more gaze alternation and food-directed showing when owners had not observed the location of hidden food and modified their behavior according to whether the human partner behaved cooperatively or competitively. The authors interpreted these findings as being consistent with flexible adjustment of showing behavior according to characteristics of the receiver and discussed these observations within the framework of intentional communication.

The expression of social signals is further modulated by developmental and endocrine processes. Castaneda et al. experimentally examined testosterone-dependent agonistic behavior in male Syrian hamsters before and after adolescence. Testosterone increased attacks and dominance in prepubertal males but not in adults, whereas testosterone-associated flank marking was observed only in adults. These findings show that behavioral responsiveness to testosterone varies across developmental stages, indicating that endocrine regulation of social behavior changes over the course of life. Mother–offspring communication represents another relevant dimension of social behavior. Mota-Rojas, Bienboire-Frosini et al. reviewed endocrine, behavioral, maternal, neonatal, and environmental factors that may interfere with the establishment of mother–young bonding in domestic mammals. The review examines the roles of oxytocin, steroid hormones, prolactin, dopamine, maternal condition, parity, prenatal factors, and neonatal vitality in maternal care and offspring recognition. Disruption of these processes may impair bond establishment and affect neonatal survival. Wegner et al. examined suckling and allosuckling behavior in dairy calves maintained in cow–calf contact systems from a different experimental perspective. Suckling patterns varied with calf age in one of the systems evaluated, whereas the probability of allosuckling increased with age in both systems. Allosuckling was also more likely when another calf was already suckling, indicating that both developmental stage and the immediate social context influence its expression.

The studies presented in this section show that the biological meaning of animal signals depends on the age at which they are produced and received. Their interpretation therefore requires consideration of the physiological, developmental, ecological, and social conditions accompanying signal exchange.

## 3 Affective states, pain and welfare

The study of affective states in animals requires the integration of behavioral, physiological, and neurobiological evidence while acknowledging the limitations inherent in inferring subjective experiences. The contributions included in this Research Topic address this issue through different approaches, including the neurobiology of pain, emotional experiences, and behavioral indicators related to animal welfare. Pain is central to animal welfare assessment, but its evaluation cannot be based on a single indicator. Mota-Rojas, Whittaker, Coria-Avila et al. reviewed the neurobiology of acute pain and its relationship with facial expressions in domestic animals. The authors describe the neural pathways involved in nociception, the development of species-specific grimace scales, and the potential use of facial expressions as indicators of acute pain. These tools, however, should be interpreted alongside behavioral and clinical assessments. From a different approach, Mota-Rojas, Whittaker, Lanzoni et al. synthesized the available evidence on body language associated with pain in domestic mammals. Postural changes, locomotor alterations, facial expressions, and species-specific behavioral responses can provide relevant information for pain assessment when considered within the clinical context. The discussion of affective states also extends to emotional pain as a subject of scientific investigation. Mota-Rojas, Whittaker, Coria-Avila, Hernández-Avalos et al. reviewed the concept of emotional pain in animals from neurobiological, behavioral, and evolutionary perspectives. Drawing upon evidence related to social separation, grief-like responses, chronic stress, and affective neuroscience, the review highlights both the growing body of evidence supporting the study of emotional pain and the methodological challenges associated with evaluating subjective experiences in non-human animals. The authors therefore stress that interpretations should remain firmly anchored in species-specific biological and behavioral evidence.

While negative affective states have traditionally received greater attention, the Research Topic also considers physiological correlates associated with positive experiences. Steinerová et al. evaluated heart rate variability during brief play bouts in pigs. During 10-s bouts, the root mean square of successive differences between normal heartbeats (RMSSD) increased during play and immediately afterward, whereas the standard deviation of normal-to-normal intervals increased only when pigs were ambulating. The increase in RMSSD was interpreted as consistent with positive valence, although physical activity also affected heart rate variability and therefore needs to be considered when these measures are used to infer emotional state.

Affective processes are also modulated by social interactions, although their behavioral expression depends on context. Du et al. conducted a systematic review and meta-analysis of emotional contagion in rodents. Their analyses showed that pain contagion was generally associated with social approach and helping-related behaviors, whereas fear contagion was more often associated with social avoidance. These findings indicate that different affective states can be expressed through distinct social responses depending on the experimental paradigm. In another social context, Vilela et al. examined self-directed behaviors in zoo-housed Asian elephants following agonistic interactions. The study reported an increase in these behaviors after conflict, suggesting that they may provide information on post-conflict responses under the conditions evaluated. Behavior can also be examined in relation to animal welfare. Quinn et al. reviewed chewing behavior in dogs and proposed an analytical framework based on Tinbergen's Four Questions and the Five Domains Model.

Their analysis argues that chewing extends beyond a feeding-related activity and should instead be interpreted within a broader framework that incorporates motivational, cognitive, health-related, and welfare dimensions.

The developmental onset of the capacity to suffer is examined from another perspective. Mace and Knight systematically reviewed the available evidence in chick embryos and chicks and concluded that this capacity is likely to be present by embryonic day 18 and may arise as early as day 13. This estimate is based on neuroanatomical, electroencephalographic, physiological, and behavioral evidence, although methodological limitations remain in the available studies.

The studies presented in this section highlight that no single behavioral or physiological response can serve as a universal indicator of affective states. Instead, affective states are expressed in species-specific ways, for example through facial and postural changes associated with pain, social avoidance associated with fear, or increased self-directed behaviors following agonistic interactions.

## 4 Physiological and environmental assessment

Physiological responses help characterize how animals interact with their environment and respond to management practices, such as handling, environmental challenges, such as heat or cold stress, and biological demands. In the studies included in this Research Topic, physiological measurements are used to assess animal responses, but their interpretation also requires consideration of behavioral observations, environmental conditions, and species-specific biological characteristics.

Infrared thermography (IRT) is used to assess thermal responses in animals. Sousa Pinto et al. conducted a systematic review of 83 studies examining its use in the assessment of thermal comfort in cattle. The authors identified the principal thermal windows used, examined methodological factors affecting surface temperature measurements, and documented substantial variability among experimental protocols. Based on the available evidence, they concluded that IRT can serve as a complementary technique for assessing thermal status, provided that environmental conditions are considered and standardized image-acquisition protocols are applied.

Two studies in rabbits examined responses under controlled thermal conditions. Contreras-Jodar, Dalmau, Bagaria, Rendon et al. found that temperature–humidity treatments altered posture, behavior, and the space occupied by the animals. In a subsequent simulated-transport experiment, Contreras-Jodar, Dalmau, Bagaria, Barbosa-Filho et al. assessed rabbits using thermophysiological and metabolic indicators together with infrared thermography. Space allowance and container height modified these responses according to thermal conditions, while lacrimal and ear temperatures were correlated with rectal temperature.

Non-invasive endocrine biomarkers have also been used to assess physiological responses in captive and free-ranging animals. Naderi et al. evaluated fecal glucocorticoid metabolites in free-ranging brown bears and reported associations between endocrine responses and different forms of human disturbance. The authors also noted that glucocorticoid concentrations are influenced by multiple biological and environmental factors and therefore need to be interpreted within the physiological context in which they are measured. Karaer et al. quantified fecal testosterone and progesterone metabolites in captive and free-ranging mountain gazelles. Concentrations varied with sex, season, and population status, with testosterone metabolite concentrations generally higher in free-ranging animals. Environmental differences between the two populations were considered possible sources of variation in hormone metabolite excretion.

A similar message is evident in equine studies. Nowak et al. followed 41 horses from a single herd for 1 year, measuring fecal cortisol metabolites (FCMs) in relation to environmental conditions, potentially stressful events, and disease. FCM concentrations increased with ambient temperature and after a riding exam and were also elevated during some painful conditions, such as traumatic injuries; however, responses varied among individuals and were not consistent across disease categories. The authors considered FCMs capable of reflecting some stress responses but insufficiently reliable for early disease detection.

Perhaps one of the most significant advances reflected in this section is the transition from describing animal responses to objectively characterizing the biological processes underlying them. In doing so, physiological approaches are broadening current understanding of the mechanisms that shape communication, behavior, and adaptation across diverse biological contexts.

## 5 Emerging methodologies and translational applications

This Research Topic includes several methodological developments applied to the study of animal behavior, communication, and welfare. Although the contributions address different species and biological questions, several seek to improve the objectivity, reproducibility, and applicability of research through technological tools, standardized procedures, and new conceptual approaches. Artificial intelligence is one of the methodological strategies represented in this section.

Martvel et al. developed automated facial landmark-based pipelines for cephalic-type, breed, and pain recognition in cats. The best models using automatically detected landmarks achieved 79% accuracy for cephalic-type recognition and 66% for pain recognition, whereas breed recognition showed lower performance, with a maximum accuracy of 48%. Performance declined when manually identified landmarks were replaced by automated detection and also varied among breeds and cephalic types. Pearline et al. applied machine learning to classify emotional states from cat and dog vocalizations, including sadness and anger in dogs, and anger and happiness in cats, using a deep-learning model that combines long short-term memory and gated recurrent unit architectures. Within the datasets evaluated, QuadEmoNet achieved 95% accuracy for dog vocalizations and 90% for cat vocalizations. Other contributions in the Research Topic address methodological development through non-computational procedures. Rojas-Guevara et al. developed standardized procedures for selecting and training wildlife-detection dogs, preparing species-specific scent materials, and certifying canine teams. Discrimination between target and distractor odors was evaluated through double-blind testing, with sensitivity, specificity, and evaluator agreement used to characterize performance.

New conceptual approaches were also proposed for behavioral assessment. Kim introduces a non-separation protocol for evaluating attachment organization in dogs. By avoiding repeated owner–dog separations, the method is intended to examine attachment-related behavior under more natural interaction conditions and is presented as a complementary alternative to existing attachment assessments. Diehl and Bader propose the Progressive Structural and Functional Change (PSF) framework to describe relationships among structural configuration, locomotor coordination, and functional change in horses. Proposed markers of PSF– include an open lumbosacral joint and temporal dissociation of the diagonal limbs during trot. These proposed markers remain to be empirically validated.

Methodological standardization is also relevant to clinical assessment. Lane and Haussler surveyed veterinarians on the perceived prevalence of back pain and dysfunction in dogs and horses. Responses varied widely according to educational background and examination methods. The authors cautioned that these perceptions do not represent true prevalence and emphasized the need for objective methods and standardized spinal examination procedures.

The studies in this section use different approaches to improve the measurement and interpretation of animal behavior and communication. Their contributions include analytical tools, standardized procedures, and assessment frameworks intended to increase the reproducibility and scientific utility of the observations.

## 6 Concluding remarks

The studies included in this Research Topic show that animal communication is shaped by interactions among neural processes, endocrine regulation, physiology, sensory perception, behavior, and environmental conditions. Signals therefore acquire meaning within the biological and ecological systems in which they are produced and received and cannot be adequately interpreted as isolated events. The study of animal communication also depends on combining information obtained at different levels of biological organization. Computational methods, emerging technologies, and physiological measurements are particularly useful when they allow to be interpreted in relation to the biological and ecological context in which it occurs. The contributions included in this eBook show how such interdisciplinary work can inform research on animal communication and related questions in animal welfare, veterinary medicine, conservation, and comparative biology.
